# Application of Risks Scores in Acute Coronary Syndromes. How Does ProACS Hold Up Against Other Risks Scores?

**DOI:** 10.5935/abc.20190109

**Published:** 2019-07

**Authors:** Júlio Gil, Luís Abreu, Hugo Antunes, Maria Luísa Gonçalves, Maria Inês Pires, Luís Ferreira dos Santos, Carla Henriques, Ana Matos, José Costa Cabral, Jorge Oliveira Santos

**Affiliations:** 1 Hospital de São Teotónio, Viseu - Portugal; 2 Instituto Politécnico de Viseu e CI&DETS, Viseu - Portugal; 3 Centro de Matemática da Universidade de Coimbra (CMUC), Coimbra - Portugal

**Keywords:** Acute Coronary Syndrome/prognosis, ST Elevation Myocardial Infarction, Hospital Mortality, Risk Assessment/methods, Survival Rate/methods

## Abstract

**Background:**

Multiple risk scores (RS) are approved in the prediction of worse prognosis in acute coronary syndromes (ACS). Recently, the Portuguese Journal of Cardiology has proposed the ProACS RS.

**Objective:**

Application of several validated RS, as well as ProACS in patients, admitted for ACS. Evaluation of each RS's performance in predicting in-hospital mortality and the occurrence of all-cause mortality or non-fatal ACS at one-year follow-up and compare them to the ProACS RS.

**Methods:**

A retrospective study of ACS was performed. The following RS were applied: GRACE, ACTION Registry-GWTG, PURSUIT, TIMI, EMMACE, SRI, CHA2DS2-VASc-HS, C-ACS and ProACS. ROC Curves were created to determine the predictive power for each RS and then were directly compared to ProACS.

**Results:**

The ProACS, ACTION Registry-GWTG and GRACE showed a c-statistics of 0.908, 0.904 and 0.890 for predicting in-hospital mortality, respectively, performing better in ST-segment elevation myocardial infarction patients. The other RS performed satisfactorily, with c-statistics over 0.750, apart from the CHA2DS2-VASc-HS and C-ACS which underperformed. All RS underperformed in predicting worse long-term prognosis revealing c-statistics under 0.700.

**Conclusion:**

ProACS is an easily obtained risk score for early stratification of in-hospital mortality. When evaluating all RS, the ProACS, ACTION Registry-GWTG and GRACE RS showed the best performance, demonstrating high capability of predicting a worse prognosis. ProACS was able to demonstrate statistically significant superiority when compared to almost all RS. Thus, the ProACS has showed that it is able to combine simplicity in the calculation of the score with good performance in predicting a worse prognosis.

## Introduction

Cardiovascular disease is the most common cause of death worldwide.^1,2^ In the past three to four decades, studies have shown a significant reduction in acute and long-term mortality by acute coronary syndromes (ACS).^1-3^ This is attributed to improvements in medical therapy and invasive strategies.^1-3^ However, ACS represent a heterogeneous group, with varying risk of morbimortality.^1-4^ Several risk stratification models have been developed to determine which patients carry a higher probability of worst outcome.^3-13^ Early risk stratification is crucial to ensure a tailored approach to each individual patient, weighing both the risks and benefits of each treatment option.^1,2^

Recently, there has been a systematic approach to risk assessment, with the creation of a myriad of risks scores (RS).^3-13^ Perhaps, the Global Registry of Acute Coronary Events (GRACE) Risk Score^6^ is the most widely recognized RS in ACS. According to the most recent European guidelines, the GRACE risk score is recommended when stratifying risk in ST-segment elevation myocardial infarction (STEMI) and non-ST-segment elevation myocardial infarction, and/or unstable angina (NSTEMI/UA).^1,2^ However, there are several other known RS, such as: the Thrombolysis in Myocardial Infarction (TIMI) for STEMI;^7^ the Platelet glycoprotein IIb/IIa in Unstable angina: Receptor Suppression Using Integrilin Therapy (PURSUIT);^5^ the Simple Risk Index (SRI);^8^ the Evaluation of the Methods and Management of Acute Coronary Events (EMMACE);^9^ and more recently, the Canada Acute Coronary Syndrome (C-ACS),^10^ the CHA_2_DS_2_-VASc-HS score,^11^ and the ACTION (Acute Coronary Treatment and Intervention Outcomes Network) Registry-GWTG.^12^

In 2016, the Portuguese Journal of Cardiology published a new risk score, formulated using the Portuguese Registry on Acute Coronary Syndromes. The Portuguese Registry of Acute Coronary Syndromes was established in 2002,^14^ under the auspice of the Portuguese Society of Cardiology. It is an observational, multicentric, nationwide prospective study in which each hospital participates with data from all patients admitted for ACS. The group developed the simple but effective ProACS risk score for predicting in-hospital mortality, which can be easily applied even in a pre-hospital setting.^3^

The objective of this article is to calculate all of the RS in patients admitted for ACS, in a single-centre study. The authors evaluate each RS's performance in predicting in-hospital mortality and compare them specifically to the ProACS RS. The authors also determine each RS's performance at predicting worse outcome in STEMI and NSTEMI/UA independently. Finally, the authors access each RS´s ability to predict mortality and recurring ACS at one-year follow-up.

## Methods

This is a retrospective study of patients admitted for ACS to a Coronary Care Unit of a centralized hospital, from December 2006 to May 2016. Only patients presenting with a history of chest pain at rest or other symptoms suggestive of an ACS with or without new significant ST-segment or T-wave changes, new left bundle branch block or elevated biomarkers of myocardial damage were included. Of the 1714 patients included in the study period, 1452 were selected, with the remaining patients being excluded due to missing data. The population sample of this study was not included in the development cohort used to formulate the ProACS risk score,^3^ although it has been included in the validation cohorts.

The following RS were calculated for all patients: GRACE, TIMI for STEMI, PURSUIT, SRI, EMMACE, C-ACS, CHA_2_DS_2_-VASc-HS, ACTION Registry-GWTG and ProACS. All RS were calculated using data from the initial clinical history, electrocardiogram and laboratory values collected on admission. All patients included were followed up for at least one year or until the occurrence of a major event. The primary endpoint of this study was in-hospital mortality and the combination of all-cause mortality or non-fatal ACS at one-year follow-up.

### Statistical analysis

Categorical variables were characterized by percentages. Group comparisons, with respect to these variables, were performed through chi-square or Fisher's exact test. Numeric continuous variables were expressed as mean ± standard deviation and RS as median with interquartile range, given their ordinal nature. Group comparisons were achieved through the Mann-Whitney test since the normality assumption was not satisfied for any of the studied numeric variables. Comparative analyses were carried out in relation to demographic variables, therapeutic strategies and general outcome parameters. The RS was evaluated by receiver operating characteristic (ROC) curves, and their area under the curve (AUC), with respect to their ability to differentiate patients with and without adverse clinical events, regarding in-hospital mortality and the combination of all-cause mortality or non-fatal ACS at one-year follow-up. The comparison of AUCs, for each RS with ProACS, was done by the method described by DeLong et al.^15^ The Hosmer-Lemeshow Test^16^ was used to evaluate the goodness of fit for each risk score. Two-sided *p*-values are reported and a p-value < 0.05 was considered statistically significant. Statistical analysis was performed with SPSS version 17.0 (SPSS Inc., Chicago, IL, USA) and MedCalc version 18.2.1 (MedCalc Software, Osted, Belgium).

## Results

### Baseline characteristics and univariate predictors of worse outcome

A total of 1,452 patients were included in this study. The baseline characteristics are displayed in Table 1. Regarding in-hospital mortality, 6.5% of the patients died. At one-year follow-up, 9.9% of the patients either died or suffered a non-fatal ACS.

**Table 1:** Characterization of the population (n=1,452)

Male gender, %	70%
**Age, years**	69.09 ± 13.2
**Type of ACS**	
STEMI	45.1%
NSTEMI/UA	52.0%
ACS with left bundle branch block	2.3%
ACS with pacing rhythm	0.6%
Systolic Blood Pressure at admission, mmHg	140.54 ± 30.4
Diastolic Blood Pressure at admission, mmHg	81.79 ± 17.7
Heart rate, beats per minute	79.29 ± 21.1
**Killip-Kimbal class at admission**	
I	70.7%
II	22.0%
III	5.0%
IV	2.3%
**Maximum Killip-Kimbal class**	
I	57.2%
II	27.3%
III	6.0%
IV	9.4%
**Risk Factors**	
Hypertension	65.8%
Dyslipidaemia	46.6%
Smoking habits	24.3%
Diabetes mellitus	26.6%
Previous known coronary disease	19.5%
Chronic kidney disease	9.7%
Cerebrovascular disease	9.4%
**Previous medication**	
Statin	35.0%
iRAAS	48.1%
Beta-blocker	17.5%
Antiplatelet therapy	34.4%
**Laboratory values**	
Hemoglobin, g/dL	13.95 ± 2.5
Creatinine, mg/dL	1.20 ± 1.6
High sensitivity troponin I at admission, ng/dL	15.92 ± 49.7
Maximum troponin I, ng/dL	69.68 ± 104.7
Brain Natriuretic Peptide, pg/dL	552.58 ± 708.0
**Medication and therapeutic strategy during hospitalization**	
iRAAS	81.9%
Beta-blocker	59.6%
Nitrates	32.4%
Antiarrhythmics	13.6%
Inotropes	12.3%
Invasive strategy	79.9%
Left Ventricular Ejection Fraction, %	53.80 ± 12.3
Hospitalization days	7.3 ± 5.0
**Risk Scores**	
TIMI for STEMI	5 (3-7)
PURSUIT	13 (10-14)
SRI	26.04 (17.82 - 37.24)
GRACE	144 (112-178.75)
EMMACE	0.15 (0.06 - 0.33)
CHA_2_DS_2_-VASc-HS	4 (3-5)
ACTION Registry-GWTG	34 (27-44)
C-ACS	1 (1-1)
ProACS	2 (1-3)
In-hospital Death	6.5%
All-cause mortality and non-fatal ACS at 1-year follow-up	9.9%

ACS: acute coronary syndrome; STEMI: ST-segment elevation myocardial infarction; NSTEMI/UA: non-ST-segment elevation myocardial infarction/ unstable angina; iRAAS: renin angiotensin aldosterone system inhibitors. TIMI: Thrombolysis in Myocardial Infarction; PURSUIT: Platelet glycoprotein IIb/IIa in Unstable angina: Receptor Suppression Using Integrilin Therapy; SRI: Simple Risk Index; GRACE: Global Registry of Acute Coronary Events; EMMACE: Evaluation of the Methods and Management of Acute Coronary Events; C-ACS: Canada Acute Coronary Syndrome. Chronic kidney disease defined as reduction of glomerular filtration rate of under 60 ml/min/1.73 m^2^.

Table 2 displays the univariate predictors for in-hospital mortality and for all-cause mortality and non-fatal ACS at one-year follow-up. Regarding in-hospital mortality, it is evident that older patients have higher mortality, with chronic kidney disease being associated with a worse prognosis. The clinical presentation also influences the outcome. Lower blood pressure and higher heart rate, as well as higher Killip-Kimball (KK) class, were linked to a higher mortality rate. It is also evident that lower haemoglobin and higher creatinine, troponin and brain natriuretic peptide values is associated with a worse prognosis, as well as a lower left ventricular ejection fraction. Regarding the occurrence of events at one-year, follow-up older and female patients tend to have higher mortality. NSTEMI/UA is associated with a worse prognosis. Lower diastolic blood pressure, a higher heart rate and KK class are associated with higher rate of events. Concerning past medical history, diabetes mellitus, chronic kidney disease and previous known coronary disease are associated with a worse outcome. A higher rate of events at 1-year follow-up was seen in patients medicated previously to the index event with statin, renin-angiotensin-aldosterone system inhibitors, beta-blocker and antiplatelet therapy. All RS scored significantly higher in the groups with worse outcome, both in in-hospital mortality and at one-year follow-up.

**Table 2:** Univariate predictors of worse prognosis

	In-Hospital Mortality			1-year Follow-up		
	With events (n = 94)	Without events (n = 1358)	p-value	With events (n = 135)	Without events (n = 1223)	p-value
Male sex, %	70.2%	70.0%	0.97	59.3%	71.2%	0.004
Age, years	76.6 ± 10.2	68.6 ± 13.2	< 0.001	75.4 ± 12.7	67.8 ± 13.1	< 0.001
**Type of ACS**						
STEMI, %	54.3%	46.9%	0.168	31.1%	48.7%	< 0.001
NSTEMI/UA, %	45.7%	53.1%		68.9%	51.3%	
Systolic Blood Pressure at admission, mmHg	121.6 ± 30	141.9 ± 30	< 0.001	138.7 ± 31.7	142.2 ± 30	0.109
Diastolic Blood Pressure at admission, mmHg	73.2 ± 18.4	82.4 ± 17.5	< 0.001	78.6 ± 17.4	82.9 ± 17.5	0.002
Heart rate, beats per minute	83.9 ± 25.6	79.0 ± 20.7	0.02	85.6 ± 21.1	78.2 ± 20.6	< 0.001
**Killip-Kimbal class at admission**						
I	34.0%	73.3%	< 0.001	43.0%	76.6%	< 0.001
II	48.9%	20.1%		43.7%	17.5%	
III	7.4%	4.8%		10.4%	4.2%	
IV	9.6%	1.8%		3.0%	1.7%	
> I	66.0%	26.7%	< 0.001	57.0%	23.4%	< 0.001
**Maximum Killip-Kimbal class**						
I	4.3%	60.9%	< 0.001	29.6%	64.3%	< 0.001
II	12.8%	28.4%		51.9%	25.8%	
III	2.1%	6.3%		13.3%	5.5%	
IV	80.9%	4.5%		5.2%	4.4%	
> I	95.7%	39.1%	< 0.001	70.4%	35.7%	< 0.001
**Risk Factors**						
Hypertension, %	70.2%	65.5%	0.355	70.4%	65.0%	0.213
Dyslipidemia, %	41.5%	46.9%	0.308	51.9%	46.4%	0.225
Smoking habits, %	16.0%	24.9%	0.051	13,3%	26.2%	0.001
Diabetes Mellitus, %	31.9%	26.2%	0.226	35.6%	25.2%	0.009
Chronic Kidney Disease, %	17.5%	9.1%	0.015	20.4%	7.6%	<0.001
Cerebrovascular disease, %	12.5%	9.2%	0.332	11.5%	8.9%	0.368
Previous known coronary disease, %	19.1%	19.5%	0.931	34.1%	17.9%	< 0.001
More than 3 Risk Factors	34.0%	29.4%	0.339	35.6%	28.7%	0.097
**Previous Medication**						
Statin, %	36.2%	34.9%	0.803	43.0%	34.0%	0.038
iRAAS, %	55.3%	47.6%	0.015	57.0%	46.6%	0.021
Beta-blocker, %	17.0%	17.5%	0.901	25.2%	16.7%	0.014
Antiplatelet therapy, %	38.3%	34.1%	0.407	58.5%	31.4%	< 0.001
**Laboratory values**						
Hemoglobin, g/dL	13.3 ± 2.4	14.0 ± 2.5	0.006	12.8 ± 2.1	14.1 ± 2.5	< 0.001
Creatinine, mg/dL	1.56 ± 0.93	1.18 ± 1.6	< 0.001	1.58 ± 1.6	1.13 ± 1.6	< 0.001
Troponin at admission, ng/dL	34.4 ± 72.2	14.6 ± 47.5	< 0.001	23.1 ± 86.1	13.7 ± 41	0.215
Maximum troponin, ng/dL	109.8 ± 146.1	67.2 ± 101.1	0.001	66.3 ± 117.5	67.3 ± 99.3	0.021
Brain Natriuretic Peptide, pg/dL	1109.0 ± 1194.9	511.3 ± 640.2	< 0.001	972.2 ± 1052.9	441.2 ± 517.6	< 0.001
**Medication and therapeutic strategy during hospitalization**						
iRAAS	59.5%	83.7%	< 0.001	78.8%	84.3%	0.090
Beta-blocker	34.2%	61.6%	< 0.001	48.7%	63.3%	0.002
Nitrates	39.2%	31.9%	0.392	28.3%	23.3%	0.082
Antiarrhythmics	21.8%	13.0%	0.038	19.5%	12.2%	0.025
Inotropes	53.2%	9.0%	< 0.001	10.6%	8.8%	0.316
Invasive strategy	54.0%	80.6%	< 0.001	56.2%	83.4%	0.001
Left Ventricular Ejection Fraction, %	40.7 ± 15.2	54.1 ± 12.0	< 0.001	50.1 ± 12.6	54.7 ± 11.8	0.001
Hospitalization days	5.6 ± 6	7.42 ± 4.8	< 0.001	9.2 ± 5.0	7.2 ± 4.8	< 0.001
**Risk Scores**						
TIMI for STEMI	7 (5-9)	4 (2-6)	< 0.001	7 (4-8)	4 (2-6)	< 0.001
PURSUIT	15 (14-16)	12 (10-14)	< 0.001	14 (12-16)	12 (10-14)	< 0.001
SRI	38.9 (28.7-54.8)	25.2 (17.5 - 35.8)	< 0.001	36.2 (23.3-48.5)	24.2 (17.0-33.8)	< 0.001
GRACE	217 (195-249)	140 (109-171)	< 0.001	170 (142-194)	137 (107-167)	< 0.001
EMMACE	0.36 (0.23-0.55)	0.14 (0.06 - 0.31)	< 0.001	0.29 (0.13-0.48)	0.13 (0.05-0.28)	< 0.001
CHA_2_DS_2_-VASc-HS	4 (3-5) 4.28 ± 1.6	4 (3-5) 3.73 ± 1.6	0.001	4 (3-5) 4.36 ± 1.8	4 (3-5) 3.7 ± 1.6	< 0.001
ACTION Registry-GWTG	58.5 (51-66)	33 (27 -42)	< 0.001	42 (33-50)	32 (26-41)	< 0.001
C-ACS	1 (1-2)	1 (1-1)	< 0.001	1 (1-2)	1 (1-1)	0.029
ProACS	5 (4-6)	2 (1- 3)	< 0.001	3 (2-4)	2 (1-3)	< 0.001

ACS: acute coronary syndrome; STEMI: ST-segment elevation myocardial infarction; NSTEMI/UA: non-ST-segment elevation myocardial infarction/ unstable angina; iRAAS: Renin angiotensin aldosterone system inhibitors; TIMI: Thrombolysis in Myocardial Infarction; PURSUIT: Platelet glycoprotein IIb/IIa in Unstable angina: Receptor Suppression Using Integrilin Therapy; Simple Risk Index; GRACE: Global Registry of Acute Coronary Events; EMMACE: Evaluation of the Methods and Management of Acute Coronary Events; C-ACS: Canada Acute Coronary Syndrome. P-values obtained by the Mann-Whitney test for numerical variables and by chi-square or Fisher's exact test for categorical variables.

### Predictive accuracy of the risk scores

**Figure 1:**
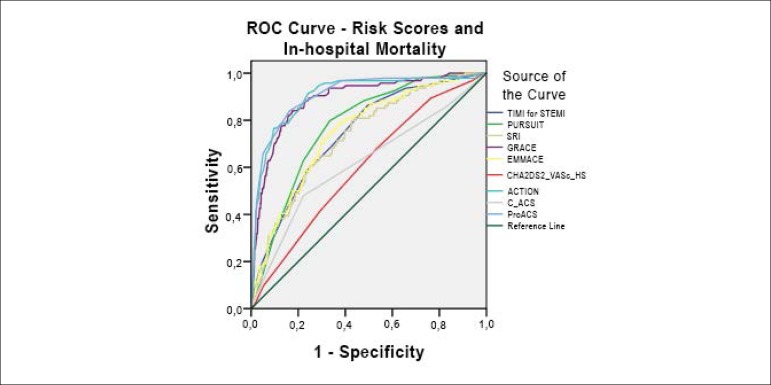
Receiver operating characteristic (ROC) curves regarding risks scores and in-hospital mortality, in the total population. TIMI: Thrombolysis in Myocardial Infarction; PURSUIT: Platelet glycoprotein IIb/IIa in Unstable angina: Receptor Suppression Using Integrilin Therapy; Simple Risk Index; GRACE: Global Registry of Acute Coronary Events; EMMACE: Evaluation of the Methods and Management of Acute Coronary Events; C-ACS: Canada Acute Coronary Syndrome.

**Figure 2:**
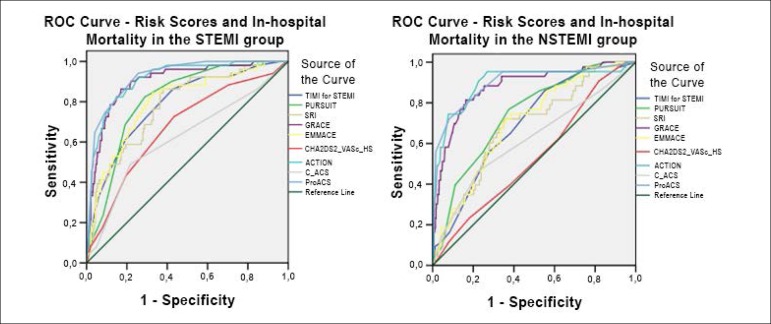
Receiver operating characteristic (ROC) curves regarding risks scores and in-hospital mortality, in the STEMI and NSTEMI population individually. TIMI: Thrombolysis in Myocardial Infarction; PURSUIT: Platelet glycoprotein IIb/IIa in Unstable angina: Receptor Suppression Using Integrilin Therapy; Simple Risk Index; GRACE: Global Registry of Acute Coronary Events; EMMACE: Evaluation of the Methods and Management of Acute Coronary Events; C-ACS: Canada Acute Coronary Syndrome.

**Figure 3:**
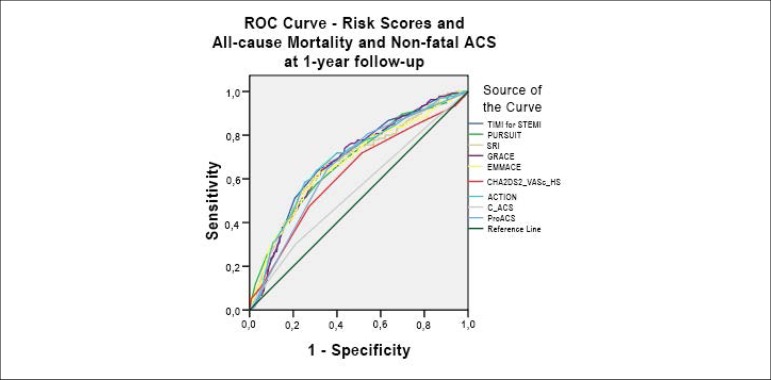
Receiver operating characteristic (ROC) curves regarding risks scores and all-cause mortality and non-fatal ACS at one-year follow-up. TIMI: Thrombolysis in Myocardial Infarction; PURSUIT: Platelet glycoprotein IIb/IIa in Unstable angina: Receptor Suppression Using Integrilin Therapy; Simple Risk Index; GRACE: Global Registry of Acute Coronary Events; EMMACE: Evaluation of the Methods and Management of Acute Coronary Events; C-ACS: Canada Acute Coronary Syndrome.

Table 3, Table 4 and Table 5 describe the predictive accuracy and goodness of fit of the RS at predicting in-hospital mortality globally, at predicting in-hospital mortality in the specific group of STEMI and NSTEMI/UA patients individually, and at predicting occurrence of all-cause mortality and non-fatal ACS at one-year follow-up, respectively. The last column of each table show how the other RS compare to the ProACS score. Figure 1 displays the ROC curves regarding the RS and in-hospital mortality. Figure 2 shows in-hospital mortality in the STEMI and NSTEMI group individually. The long-term prognosis is demonstrated in Figure 3.

**Table 3:** Predictive accuracy and goodness of fit of the scores at predicting in-hospital mortality and comparation with the ProACS risk score

	In-hospital mortality				
	c-statistics (95% CI)	p-value	p-value (Hosmer-Lemeshow χ^2^)	Comparing with the ProACS Risk Score	
				∆	p-value
TIMI for STEMI	0.744 (0.695-0.792)	< 0.001	0.486	0.165	< 0.0001
PURSUIT	0.775 (0.733-0.817)	< 0.001	0.043	0.133	< 0.0001
SRI	0.732 (0.682-0.781)	< 0.001	0.23	0.176	< 0.0001
GRACE	0.890 (0.855-0.925)	< 0.001	0,298	0.0185	0.0879
EMMACE	0.749 (0.700-0.797	< 0.001	0.566	0.160	< 0.0001
CHA_2_DS_2_-VASc-HS	0.600 (0.543-0.656)	0.001	0,804	0.309	< 0.0001
ACTION Registry-GWTG	0.904 (0.870-0.938)	< 0.001	0.041	0.00399	0.6647
C-ACS	0.619 (0.554-0.684)	< 0.001	0.003	0.289	< 0.0001
ProACS	0.908 (0.876-0.941)	< 0.001	0.031	N/A	N/A

∆: difference between the two AUC (area under the curve). TIMI: Thrombolysis in Myocardial Infarction; PURSUIT: Platelet glycoprotein IIb/IIa in Unstable angina: Receptor Suppression Using Integrilin Therapy; Simple Risk Index; GRACE: Global Registry of Acute Coronary Events; EMMACE: Evaluation of the Methods and Management of Acute Coronary Events; C-ACS: Canada Acute Coronary Syndrome.

**Table 4:** Predictive accuracy and goodness of fit of the scores at predicting in-hospital mortality and comparation with the ProACS risk score, in both STEMI and NSTEMI/UA

**STEMI**	**In-hospital mortality**				
	**c-statistics (95% CI)**	**p-value**	**p-value (Hosmer-Lemeshow χ^2^)**	**Comparing with the ProACS Risk Score**	
				**∆**	**p-value**
TIMI for STEMI	0.785 (0.720-0.849)	< 0.001	0.766	0.139	< 0.0001
PURSUIT	0.809 (0.758-0.861)	< 0.001	0.075	0.114	< 0.0001
SRI	0.781 (0.718-0.843)	< 0.001	0.011	0.143	< 0.0001
GRACE	0.899 (0.856-0.942)	< 0.001	0.603	0.0244	0.0331
EMMACE	0.795 (0.731-0.858)	< 0.001	0.392	0.129	< 0.0001
CHA_2_DS_2_-VASc-HS	0.674 (0.596-0.751)	< 0.001	0.206	0.250	< 0.0001
ACTION Registry-GWTG	0.911 (0.874-0.948)	< 0.001	0.882	0.0127	0.2248
C-ACS	0.620 (0.531-0.708)	0.004	0.005	0.304	< 0.0001
ProACS	0.923 (0.892-0.955)	< 0.001	0.821	N/A	N/A
**NSTEMI/UA**	**c-statistics (95% CI)**	**p-value**	**p-value (Hosmer-Lemeshow χ^2^)**	**Comparing with the ProACS Risk Score**	
				**∆**	**p-value**
TIMI for STEMI	0.696 (0.624-0.767)	< 0.001	0.377	0.202	< 0.0001
PURSUIT	0.742 (0.673-0.810)	< 0.001	0.551	0.157	< 0.0001
SRI	0.682 (0.604-0.761)	< 0.001	0.078	0.216	< 0.0001
GRACE	0.878 (0.822-0.934)	< 0.001	0.566	0.0205	0.2040
EMMACE	0.702 (0.629-0.774)	< 0.001	0.376	0.197	< 0.0001
CHA_2_DS_2_-VASc-HS	0.534 (0.448-0.620)	0.453	0.455	0.364	< 0.0001
ACTION Registry-GWTG	0.895 (0.835-0.956)	< 0.001	< 0.001	0.00302	0.8411
C-ACS	0.618 (0.522-0.714)	0.009	0.077	0.281	< 0.0001
ProACS	0.898 (0.841-0.956)	< 0.001	0.001	N/A	N/A

∆: difference between the two AUC (area under the curve). TIMI: Thrombolysis in Myocardial Infarction; PURSUIT: Platelet glycoprotein IIb/IIa in Unstable angina: Receptor Suppression Using Integrilin Therapy; Simple Risk Index; GRACE: Global Registry of Acute Coronary Events; EMMACE: Evaluation of the Methods and Management of Acute Coronary Events; C-ACS: Canada Acute Coronary Syndrome.

**Table 5:** Predictive accuracy and goodness of fit of the scores at predicting the occurrence of all-cause mortality and non-fatal ACS at one‑year follow-up and comparation with the ProACS risk score

	All-cause mortality and non-fatal ACS at one-year follow-up				
	c-statistics (95% CI)	p-value	p-value (Hosmer-Lemeshow χ^2^)	Comparing with the ProACS Risk Score	
				∆	p-value
TIMI for STEMI	0.695 (0.650-0.741)	< 0.001	0.033	0.0323	0.0656
PURSUIT	0.682 (0.634-0.730)	< 0.001	0.001	0.0185	0.3846
SRI	0.680 (0.632-0.729)	< 0.001	0.042	0.0171	0.3854
GRACE	0.684 (0.639-0.729)	< 0.001	0.022	0.0209	0.1608
EMMACE	0.673 (0.623-0.723)	< 0.001	0.681	0.00997	0.6157
CHA_2_DS_2_-VASc-HS	0.622 (0.570-0.673)	< 0.001	0.027	0.0414	0.2093
ACTION Registry-GWTG	0.690 (0.643-0.737)	< 0.001	0.005	0.0267	0.0567
C-ACS	0.550 (0.497-0.603)	0.057	0.366	0.113	0.0007
ProACS	0.663 (0.617-0.709)	< 0.001	0.015	N/A	N/A

∆: difference between the two AUC (area under the curve). TIMI: Thrombolysis in Myocardial Infarction; PURSUIT: Platelet glycoprotein IIb/IIa in Unstable angina: Receptor Suppression Using Integrilin Therapy; Simple Risk Index; GRACE: Global Registry of Acute Coronary Events; EMMACE: Evaluation of the Methods and Management of Acute Coronary Events; C-ACS: Canada Acute Coronary Syndrome.

The majority of the RS showed a good discriminatory accuracy to predict in-hospital mortality, as demonstrated by c-statistics consistently over 0.700. Notably, three RS outperformed the others, namely the GRACE, ACTION Registry-GWTG and ProACS RS, with c-statistics around 0.900. Most RS, apart from the ProACS (p = 0.031), PURSUIT (p = 0.043), ACTION Registry-GWTG (p = 0.041) and C-ACS (p = 0.003) RS, showed an adequate fit, as demonstrated by a p-value for the Hosmer-Lemeshow (HL) test over 0.05. Comparing ProACS to the other RS revealed a statistically significant superiority of the first to all except the ACTION Registry-GWTG (p = 0.6647) and the GRACE (p = 0.0879).

All RS consistently showed better discriminatory accuracy at predicting in-hospital mortality in STEMI patients. In this population, the ACTION Registry-GWTG and ProACS RS performed incredibly well, with c-statistics of over 0.900. Almost all RS revealed an adequate fit, except for the SRI (p = 0.011), the C-ACS (p = 0.005) and a trend from PURSUIT (p = 0.075). In STEMI patients, the ACTION Registry-GWTG (p = 0.882) and ProACS RS (p = 0.821) showed good fit. The ProACS RS demonstrated statistically significant superior discriminatory accuracy when compared to all other RS, except for ACTION Registry-GWTG (p = 0.2248).

In the NSTEMI population, the RS performed slightly worse when compared to STEMI patients. ProACS, ACTION Registry-GWTG and GRACE scores were the RS with the highest discriminatory accuracy at predicting in-hospital mortality, with c-statistics of 0.898, 0.895 and 0.878 respectively. ProACS also demonstrated significant superiority, except when compared to the aforementioned RS. However, the ProACS and ACTION Registry-GWTG presented with a HL test p-value of 0.001 and < 0.001, respectively, indicating model lack of fit.

Regarding all-cause mortality and non-fatal ACS at one-year follow-up, all RS underperformed, with c-statistics consistently under 0.700. ProACS was only statistically superior to the C-ACS RS, which showed particularly poor discriminatory accuracy (c-statistic 0.550). Most RS revealed model lack of fit.

## Discussion

### Development of risks scores in acute coronary syndromes

Advances in medical therapy and the development of invasive strategies has had a significant impact on prognosis in ACS.^1-3^ Risk stratification has become an essential part of the establishment of a personalized treatment strategy in patients with ACS, weighing the risks and benefits of an early invasive approach.^1,2^ In STEMI patients, primary percutaneous coronary intervention is the standard approach, thus early risk stratification is less important.^1,3^ However, risk stratification in STEMI still plays an important role in predicting which patients are at higher risk for mortality or recurrent ACS, thus warranting a more aggressive medical therapy.^1^ Patients with NSTEMI/UA represent a much more heterogeneous group, with early risk stratification playing a more central role in deciding which patients benefit more from an early invasive strategy.^2-5,13^ Several risk score have been formulated in the last 20 years, attempting to best predict which patients are at a higher risk for a worse outcome.^3-13^ The simple TIMI risk score for STEMI^7^ and for NSTEMI/UA^13^ was developed from large clinical trials, with controlled and selected populations. The TIMI RS for STEMI^7^ was formulated from the InTIME II trial which enrolled a total of 15,078 patients, all were candidates for fibrinolytic therapy. This risk score performed well at identifying high risk patients (c-statistics for predicting in-hospital mortality and in the first 24 hours after admission was 0.784 and 0.813, respectively).^7^ The TIMI for NSTEMI/UA was developed using the database of the TIMI 11B trial, with a total of 3910 patients, satisfactorily predicting all-cause mortality, myocardial infarction or urgent revascularization at 14 days.^13^ However, since it underperformed in our population, the authors decided not to use the RS. The SRI risk score was also calculated from the InTIME trial, using a cohort of 13,253 STEMI patients. This risk score satisfactorily predicted in-hospital death (c-statistic 0.79). The PURSUIT risk score was developed through the Platelet glycoprotein IIb/IIIa in Unstable angina: Receptor Suppression Using Integrilin (eptifibatide) Therapy trial using a NSTEMI population of 9,461 patients, with a c-statistic of 0.814. These RS are simple and intuitive, however, derivation from large trial databases tend to overlook specific high-risk patients.^3-5^ The GRACE risk score was developed using an international registry, much more representative of real-world patients, with a total of 11,389 patients enrolled.^6^ The GRACE risk score outperformed previous RS which tended to use clinical trial data. GRACE showed good predictive capacity for in-hospital mortality and at 6-month follow-up.^6,17^ This risk score was updated using a cohort of 48,023 patients^18^ and has become the most widely used risk score both in STEMI and NSTEMI/UA.^1,2^ The EMMACE risk score was also developed from patients admitted for ACS over a 3-month period in 1995, compiling a total of 2,135 patients.^9^

A mathematical formula only comprising 3 variables (age, heart rate and systolic blood pressure) was formulated and revealed good performance at predicting mortality at 30 days (c-statistics of 0.76 to 0.79). This risk score is simple and reproducible.^9^

The Journal of the American College of Cardiology published in 2016 a new risk score.^11^ This risk score was developed using data from the ACTION Registry-GWTG, which included a total of 145,952 patients from more than 300 hospitals from the United States of America admitted for both STEMI and NSTEMI.^11^ The ACTION Registry-GWTG risk score performed well in the general population (c-statistic 0.88), as well as in specific subsets of patients.^11^ This score appeared to be a good alternative to the GRACE score.

Finally, in 2017, the Portuguese Journal of Cardiology presented a new and simple risk score.^3^ The ProACS risk score, formulated by Timóteo et al.^3^ was developed using the Portuguese Registry of Acute Coronary Syndromes. The risk model was developed from the data of 17,380 patients. Internal and external validation of the score was done using 12,701 and 8,532 patients, respectively.^3^ Timóteo et al.^3^ built a simple risk score with only 4 variables, age, systolic blood pressure, Killip class and ST-segment elevation (information easily obtainable even in a pre-hospital setting). The score performed well in predicting in-hospital mortality, both in STEMI and NSTEMI (c-statistics ranging from 0.785 to 0.809). This risk score was formulated similarly to the C-ACS, a score formulated by a Canadian group and published in the American Heart Journal. The C-ACS developed a simple score with 4 variables (age ≥75, Killip class >I, systolic blood pressure < 100 mmHg and heart rate > 100 beats/min.^10^ The score was derived from the Acute Myocardial Infarction in Quebec (AMI-QUEBEC) and Canada ACS-1 registries, compiling a total of 6,182 patients.^10^ This score performed well at predicting worse outcome both in short-term (c-statistics ranging from 0.73 to 0.75) and in long-term mortality (c-statistics ranging from 0.73 to 0.76).^10^

It was the authors´ objective to test several RS, which have been validated in the setting of ACS, to determine which one fared better at balancing a good predictive capability, combined with simple and intuitive use. The authors decided to apply the aforementioned RS in a single-centre population of patients admitted for ACS and compare each score to the ProACS.

### Risk scores and in-hospital mortality

Almost all RS performed well. However, the CHA_2_DS_2_-VASc-HS and the C-ACS scores underperformed in this population (c-statistics of 0.600 and 0.619, respectively), even though both have been validated for the prediction of short-term mortality. The TIMI for STEMI, PURSUIT, SRI and EMMACE RS performed moderately well, with a c-statistics of 0.744, 0.775, 0.732 and 0.749, respectively. Of all the RS, three outperformed the other, achieving extremely good c-statstics, namely the ProACS, GRACE and ACTION Registry-GWTG RS. All of the RS predict short-term mortality. However, not all are equally efficient. The ProACS, GRACE and ACTION Registry-GWTG RS performed incredibly well when determining short-term mortality. A c-statistics of 0.908, 0.904 and 0.890 was calculated for each respective score. These results demonstrate a greater efficiency that that shown in previous studies.^3,6,12,18^ The ProACS demonstrated impressive results. It was significantly better than all the other RS, apart from the ACTION Registry-GWTG and GRACE RS. The only setback was a HL-test value of under 0.05 in both the ProACS and ACTION Registry-GWTG, indicating model lack of fit. This resulted from the presence of NSTEMI patients in the study.

All RS performed better at predicting in-hospital mortality in a STEMI setting. Again, the ProACS, ACTION Registry-GWTG and GRACE RS were the more accomplished RS, attaining c-statistics of 0.923, 0.911 and 0.899, respectively. These numbers are especially impressive since they outperformed each of their derivation and validation cohorts.^3,12,18^ ProACS demonstrated statistical superiority when compared to all others RS, apart from the ACTION Registry-GWTG and only marginal superiority when compared to the GRACE RS. In STEMI patients, these three RS revealed good fit. Once more, the TIMI for STEMI, PURSUIT, SRI and EMMACE RS had a satisfactory performance (c-statistics of 0.785, 0.809, 0.781 and 0.795, respectively). The CHA_2_DS_2_-VASc-HS and the C-ACS RS performed disappointingly, with c-statistics of 0.674 and 0.620, respectively.

Concerning NSTEMI, the ProACS, GRACE and ACTION Registry-GWTG achieved a good predictive power, with c-statistics of 0.898, 0.878 and 0.895. In this particular population, both the ProACS and the ACTION Registry-GWTG showed lack of fit, thus interfering with the goodness of fit in the general population in these RS. The PURSUIT and EMMACE RS performed moderately good, with c-statistics of 0.742 and 0.702. It is impressive that the PURSUIT RS performed better at predicting a worse outcome in STEMI when compared to NSTEMI, since it is based upon NSTEMI patients.^4,5^ The TIMI for STEMI and SRI predictably underperformed (c-statistics of 0.696 and 0.682), since both were developed for STEMI patients.^7^ Again, the C-ACS revealed poor discriminatory accuracy (c-statistic 0.618) and the CHA_2_DS_2_-VASc-HS was unable to predict in-hospital mortality in NSTEMI patients (c-statistic of 0.534, p = 0.453).

### Risk scores and long-term prognosis

The majority of the RS evaluated were developed solely for prediction of short-term prognosis.^3-13^ In this population, all the RS underperformed when predicting all-cause mortality and non-fatal ACS at one-year follow-up (c-statistics < 0.7). Almost all the RS presented with a c-statistic ranging from 0.622 to 0.690, without a statistically significant difference when compared with the ProACS. Notably, the C-ACS was unable to predict the worst long-term prognosis (c-statistic 0.550, p = 0.057), even though it was validated for long-term prognosis prediction.^10^ More studies are needed to develop RS with better discriminatory accuracy for predicting long-term prognosis in ACS patients.

### Limitations

This is a single-centre retrospective, observational study of a small population. The analysis of the parameters was based on nonrandomized data. The population sample was relatively small and was composed by the sequential patients admitted in a single centralized hospital, thus it might represent a biased sample.

## Conclusions

In this population, several RS showed good discriminatory accuracy at predicting short-term mortality. The ProACS, GRACE and ACTION Registry-GWTG RS performed incredibly, with c-statistics around 0.90. This revealed great predictive capability both in STEMI and NSTEMI patients. The TIMI for STEMI, PURSUIT, SRI and EMMACE RS performed moderately well. However, the CHA_2_DS_2_-VASc-HS^20^ and the C-ACS underperformed, perhaps due to differences between the cohort from which they were based on and the population sample of this study. In this real-world population, it is evident that RS developed from databases of large registries, such as the GRACE, ProACS and the ACTION Registry-GWTG, seem to fare better than those derived from clinical trials. RS developed from clinical trials tend to include skewed populations which avoid high-risk patients. None of the RS performed well at predicting long-term prognosis. This is understandable given they were intended for the prediction of short-term mortality.

The ProACS risk score proved to be an effective risk model, which performed incredibly well in this population, in both STEMI and NSTEMI patients. It is an intuitive risk score that requires only four easily obtainable variables. Its simplicity is rivalled only by the C-ACS, which has significantly underperformed in every aspect. The authors believe that ProACS is an appropriate and simple method to obtain adequate risk stratification regarding short-term prognosis that applies well to the Portuguese population.
